# Mortality from Cancers of the Digestive System among Grand Multiparous Women in Taiwan

**DOI:** 10.3390/ijerph110404374

**Published:** 2014-04-22

**Authors:** Brian K. Chen, Chun-Yuh Yang

**Affiliations:** 1 Department of Health Services Policy and Management, Arnold School of Public Health, University of South Carolina, Columbia, SC 29208, USA.; E-Mail: bchen@mailbox.sc.edu; 2 Department of Public Health, College of Health Sciences, Kaohsiung Medical University, Kaohsiung 807, Taiwan; 3 Division of Environmental Health and Occupational Medicine, National Health Research Institute, Miaoli 350, Taiwan

**Keywords:** digestive system, grand multiparous, standardized mortality ratio

## Abstract

The aim of this study is to evaluate the significance of grand multiparous (GM) status in the mortality from cancers of the digestive system among a cohort of GM women in Taiwan during the period 1978–2008. The study cohort consisted of 144,922 women with at least five children (GM women) in the Taiwan Birth Register between 1 January 1978 and 31 December 2003. Standardized mortality ratios (SMRs) for cancers of the digestive system including esophagus, stomach, colon, rectum, liver, and pancreas were calculated by dividing the numbers of observed cancer deaths to the expected numbers of deaths based on the rates of national female population. Among the 144,922 GM women, a total of 23, 220, 213, 92, 397, and 65 deaths were caused by cancers of the esophagus, stomach, colon, rectum, liver, and pancreas, respectively. The SMRs among GM women were 1.61 (95% confidence intervals (CI): 0.95–2.27) for esophageal cancer, 1.15 (95% CI: 1.00–1.31) for stomach cancer, 1.07 (95% CI: 0.93–1.22) for colon cancer, 0.94 (95% CI: 0.75–1.14) for rectal cancer, 1.18 (95% CI: 1.06–1.30) for liver cancer, and 0.79 (95% CI: 0.60–0.98) for pancreatic cancer. This study provides evidence that grand multiparity may confer a protective effect on the risk of death from pancreatic cancer. However, the results suggest that GM women may increase the risk of death from cancers of the liver and stomach.

## 1. Introduction

A substantial body of experimental, clinical, and epidemiologic evidence indicates that endogenous hormones play a major role in the etiology of several human cancers, especially for hormone dependent cancers including cancers of the breast, ovary, and endometrium [1,2]. Pregnancy alters the levels of circulating hormones including estrogens, progestins and insulin-like growth factors IGF1 and IGF2 [3]. These changes may control malignant transformation of reproductive organs [3,4].

Men tend to have a higher incidence of colorectal cancer than women of a similar age [5]. The difference between male-to-female incidence rates of gastric cancer is greatest during the reproductive ages, and the rates become more similar after menopause [6]. Men have a higher risk of liver cancer than women, with a ratio of between 2:1 to 4:1 [7]. Pancreatic cancer is about 50% more common in men than in women [8]. Men have a 7- to 10-fold higher incidence of esophageal adenocarcinoma and had a male:female ratio ranged from 3 to 4 for esophageal cell carcinoma [9]. The gender-associated difference in incidence rates may be attributable to an as yet unidentified protective factor in women, such as female sex hormones [10]. It has been hypothesized that role of sex hormone in the etiology of cancers of the digestive system may be similar to that observed in hormone-related cancers (breast, endometrial, and ovary) [6,11,12,13,14]. 

The term grand multiparity (GM) has been used to denote women who have previously given birth to five or more viable pregnancies [15]. The impact of larger number of pregnancies has been insufficiently explored [4]. The aim of this study is to evaluate the significance of GM in the mortality from cancers of the digestive system among a cohort of GM women in Taiwan during the period 1978–2008.

## 2. Materials and Methods

### 2.1. Data Source

All births are compulsorily reported to the Taiwan Local Household Birth Registry, which is managed by the Ministry of Interior (MOI), within 15 days of delivery. The MOI has released computerized Birth Registration Database since 1978. The birth–related information obtained included birth date, single/multiple pregnancy, gestational age, infant gender, birth weight and parental information, including maternal age, marital status, educational levels, and maternal parity. Because most deliveries in Taiwan take place in either a hospital or clinic [16] and the birth certificates are completed by physicians attending the delivery and it is mandatory to register all live births at local household registration offices, the birth registration data are considered complete, reliable and accurate. These data have been used in our previous studies [17].

### 2.2. Study Cohort

The study cohort consisted of 144,922 women with at least five children (GM women) in the Birth Register between 1 January 1978 (date of the computerized database on live births was released) and 31 December 2003. The choice of 2003 was motivated to allow each subject to have been follow-up for at least five years (the mortality database data were available through 31 December 2008). A detailed description of this cohort has been published elsewhere [17].

### 2.3. Follow-up

Each woman has her own unique personal identification number. Using their personal identification number, we tracked each woman to 31 December 2008, and their vital status was ascertained by linking records with the computerized mortality database, identifying the date of cause of any deaths. The International Classification of Disease, Injury, and Causes of Deaths (the 8th revision in 1978–1980 and the 9th revision in 1981–2008) was used to code the cause of death. Since it is mandatory to register death certificates at local household registration offices, the mortality statistics in Taiwan were considered to be highly accurate and complete [17].

### 2.4. Statistics

The person-years of follow-up for each woman was calculated from the date of the fifth childbirth to the date of death or 31 December 2008. Standardized mortality ratios (SMRs) were calculated to compare the mortality of cancers of the digestive system among the GM women to that of Taiwan’s entire female population. SMR is the ratio of the number of observed cancer deaths in the cohort to the number of expected cancer deaths in the cohort. The expected number of cancer death was calculated by multiplying a standard set of calendar year/age–specific rates among all Taiwanese women to the number of person-years in each stratum of the GM women cohort. The 95% confidence intervals (CIs) for the SMRs were also calculated and were based on the assumption that the number of observed cancer deaths follows a Poisson distribution [18]. Analyses were performed using the SAS statistical package (version 9.2, SAS Institute Inc., Cary, NC, USA). All statistical tests were two-sided. Values of *p* < 0.05 were considered statistically significant.

## 3. Results and Discussion

A total of 3,452,977 person-years were observed among the 144,922 GM women during the follow-up period from the time of their fifth childbirth to December 31, 2008. The mean and median ages at the start of follow-up were 31.17 (SD = 4.91) and 31 years (range: 18–52 years), respectively. 48.4% of the GM women were aged 30 years or below at the start of follow–up, 28.3% were aged 31–34 years, 17.2% were aged 35–39 years, and 6.1% were aged 40 years or above. The average time of follow-up was 23.04 years (range: 0–29.92 years). Of the 144,922 GM women, 95,776 (66.1%) had five children, 30,159 (20.8%) had six children, and 18,987 (13.1%) had seven children or more. The GM women were nearly all married (96.9%) (Table 1).

By the end of 2008, among the 144,922 GM women, 8,568 deaths (all-causes of deaths) occurred, a slightly exceeded the national average (expected deaths = 6,486.10) (SMR = 1.32, 95% CI = 1.29–1.35). The SMRs were 1.61 (95% CI: 0.95–2.27) for esophageal cancer, 1.15 (95% CI: 1.00–1.31) for gastric cancer, 1.07 (95% CI: 0.93–1.22) for colon cancer, 0.94 (95% CI: 0.75–1.14) for rectal cancer, 1.18 (95% CI: 1.06–1.30) for liver cancer, and 0.79 (95% CI: 0.60–0.98) for pancreatic cancer. Significantly low mortality was found for cancer of the pancreas. However, increased mortality was found for cancers of the liver and stomach (Table 2).

**Table 1:** Demographic characteristics of the study cohort.

Variables	Number of Subjects (%)
*Age at recruitment (fifth birth)*	
≤30	70,173 (48.42)
31–34	41,054 (28.32)
35–39	24,876 (17.17)
40+	8,819 ( 6.09)
*Parity*	
5	95,776 (66.09)
6	30,159 (20.81)
7+	18,987 (13.10)
*Marital status*	
Married	140,497 (96.95)
Not Married	4,425 (3.05)
*Years of schooling*	
≤9 years	133,939 (92.42)
≥9 years	10,983 (7.58)

**Table 2:** Standardized mortality ratios of cancers due to the digestive system among grand multiparous women in Taiwan, 1978–2008.

Cancer Sites (ICD-9 codes)	Observed	Expected	SMR	95% CI
Esophagus (150)	23	14.26	1.61	0.95–2.27
Stomach (151)	220	190.74	1.15	1.00–1.31
Colon (153)	213	198.27	1.07	0.93–1.22
Rectum (154)	92	97.61	0.94	0.75–1.14
Liver (155)	397	336.13	1.18	1.06–1.30
Pancreas (157)	65	82.51	0.79	0.60–0.98

The main findings of our study were that mortality from cancers of the liver and stomach among the GM women were 18% and 15% higher than among national reference women, who on average have about two children, respectively. Mortality from pancreatic cancer was decreased by 21%.

Our study results support the hypothesis that multiparity reduces the risk of death from cancer of the pancreas [19,20,21,22]. Furthermore, it is likely that the protective effect of multiple pregnancies extends up to five or more births. Our findings of a reduced mortality from cancer of the pancreas among the Taiwan’s GM women is not in agreement with a recent Finnish study which was based on a cohort of 87,922 GM women and found the mortality among GM women was not decreased (SMR = 0.99, 95% CI: 0.89–1.08) [4]. An explanation for this discrepancy is difficult. One reason for this could be that the distribution of causes of death differs between Taiwan and Finland. To our knowledge, this is the largest cohort (n = 144,922 GM women) published to date to estimate the impact and relative importance of grand multiparity on women’s pancreatic cancer mortality and is also the first in an Asian country. 

In the present study, we found a marked elevated risk of death from gastric cancer (15% higher than the national average), which is consistent with results from previous studies [23,24,25]. Some other studies, however, did not show any association between parity and risk of gastric cancer [26,27,28,29,30,31]. Our data did not provide support for the hypothesis that estrogens confer a protective effect on the risk of gastric cancer [6]. On the other hand, it has been reported that estrogen stimulates the growth of gastric cancer cell lines [32], and there is evidence that pregnancy or delivery might accelerate the growth of gastric cancer [33]. Our finding of an increased risk of gastric cancer among GM women may plausibly be related to a short-term increase in risk after a delivery [31].

Mortality from liver cancer was 18% higher than the national average, which is in agreement with previous studies [23,34,35,36], but is not in agreement with some studies reporting decreased risk [37,38] and others reporting no association [4,39,40,41]. However, it should be noted that most previous studies examined a very limited number of case subjects, except for two studies conducted in Taiwan [37,38], making it very likely they involved inconsistent estimates as well as limited statistical power. Our data did not support the hypothesis that sex hormones play a protective role in the etiology of liver cancer and is also contrary to previous Taiwanese studies. The reasons for the varying results are unknown but may relate to methodological issues, including age at enrollment, sample size, length of follow–up and the selection of comparison group. Of the GM women, only 7.6% had a higher education level (>9 years of schooling). The respective proportion in the general female population was 44.1% [22]. There might be differences in lifestyle between GM women and general female population. For example, the prevalence of smoking is strongly associated with social class [42], with a higher prevalence rate among the lower social class groups. However, smoking is not a major risk factor for liver cancer [37] and is infrequent among women in Taiwan [42]. Because the education level was higher in the reference population than in the GM women cohort, our finding of an elevated risk of death from liver cancer among GM women might be due to the fact that women with less education are more likely to be infected with hepatitis B and C viruses, or to have aflatoxin exposure [43]. 

Overall, the SMRs for colon and rectal cancer mortality did not deviate from the national average in the present study. This finding is in accord with the results of most of the large number of studies that have addressed the association between parity and risk of colorectal cancer [44]. Our finding of an increased risk of death from esophageal cancer seems uncertain since the result was not statistically significant. Three previous studies on parity and esophageal cancer also reported no association [4,10,45]. 

Mortality data have been widely used to generate epidemiologic hypotheses, despite their inherent limitations. In the event of a death in Taiwan, the decedent’s family is required to obtain a death certificate from the hospital or local community clinic, which then must be submitted to the household registration office in order to cancel the decedent's household registration. The death certificate is required in order to have the decedent's body buried or cremated. It is also mandatory to register all deaths at local household registration offices, thus the death registration is complete. In Taiwan, the main cause of a death must be recorded by a physician on a death certificate, which is forwarded to the National Health Department. Malignant neoplasms, including cancers of the digestive system, have been reported to be one of the most unequivocally classified causes of death in Taiwan [46]. Nonetheless, the potential for misclassification cannot be ruled out. However, any potential misclassification may be non-differential (the misclassification is unlikely to be related parity), the observed estimates in the study may be attenuated or biased towards the null. Therefore it would be unlikely to introduce a bias regarding the comparison between GM women and the reference population. The complete population coverage and follow–up made possible by the national identification number has left the study without selection bias. 

As mentioned, of the GM women, only 7.58% had a higher education level (>9 years of schooling), while the respective proportion in the general female population was 44.10% [22]. However, it is unlikely that the education level could have influenced the physician to fill in death information given on the death certificate because a physician’s reporting should be based on his or her professional knowledge and experience.

No information was available in our study on the use of oral contraceptive and hormone replacement therapy (HRT), which may have impacted on the risk of digestive cancers. We were unable to adjust for these two factors in the current study due to the lack of available data. However, the use of oral contraceptive and HRT is uncommon among Taiwanese women in the age range that encompasses the majority of women in this study [17] and is thus unlikely to have biased the results. 

There is unfortunately no information available on other variables which are likely to be related to cancers of the esophagus (such as tobacco smoking and alcohol use), stomach (such as Helicobacter pylori infection), colon (such as physical activity and dietary habits), rectum (such as physical activity and dietary habits), liver (such as hepatitis B or C infection), and pancreas (such as tobacco smoking). It seems likely that the above-mentioned factors are associated with social class (*i.e.*, differs substantially between GM women and the reference population). Moreover, observational studies have frequently shown that within a population risk is higher among the lower socioeconomic categories. However, tobacco smoking and alcohol consumption are infrequent among women in Taiwan [47]. We think that the degree to which not controlling for these two variables may have affected our results should be small if it existed. We could not adjust for potential confounding variables (e.g., physical activity, dietary habits, Helicobacter pylori infection, and hepatitis B or C infection) because of the lack of information for individual study subjects. Because the education level was higher in the reference population than in the GM women cohort [17], our SMR estimates might be a bit too high. Nonetheless, the lack of information on potential confounding variables should be regarded as a limitation of this study. Clearly, more work will be needed to clarify the role of grand multiparity in the risk of cancers of the digestive system. 

The GM women were nearly all married (96.9%). During the 1980s (1978–1987), about 97.5% of the women with a record of a first and singleton childbirth in the birth registration system were married [22]. The frequency of marriage among GM women does not differ markedly from that of the reference population. Therefore the results of this study are unlikely to be affected remarkably by marital status. 

## 4. Conclusions

In summary, we found that mortality from cancers of the stomach and liver among GM women were higher than the national average and mortality from cancer of the pancreas was below the national average. This finding adds evidence to support the hypothesis that parturition confers a protective effect on the risk of death from pancreatic cancer. However, due to the lack of individual data on possible confounders, the independent effect of parity on the risk of death from cancers of the digestive system remains open [4].
